# Primary Immunodeficiencies and Hematologic Malignancies: A Diagnostic Approach

**DOI:** 10.3389/fimmu.2022.852937

**Published:** 2022-03-18

**Authors:** Sharat Chandra, Tatiana Kalashnikova, Nicola A. M. Wright, Blachy J. Dávila Saldaña

**Affiliations:** ^1^Division of Bone Marrow Transplantation and Immune Deficiency, Cincinnati Children’s Hospital Medical Center, Cincinnati, OH, United States; ^2^Department of Pediatrics, University of Cincinnati College of Medicine, Cincinnati, OH, United States; ^3^Department of Pediatrics, Alberta Children’s Hospital, University of Calgary, Calgary, AB, Canada; ^4^Division of Blood and Marrow Transplantation, Children’s National Hospital, Washington, DC, United States; ^5^Department of Pediatrics, George Washington University, Washington, DC, United States

**Keywords:** primary immunodeficiencies, pediatric cancer, Pediatric Immunology, Pediatric immunodeficiency syndrome, malignancy, inborn errors of immunity

## Introduction

Primary immunodeficiencies (PIDs) are a heterogeneous group of inherited disorders characterized by aberrant immune function that leads to increased susceptibility to infections and/or immune dysregulation. In addition, some PIDs have an increased predisposition to malignancy. Amongst patients enrolled in the United States Immune Deficiency Network `(USIDNET) registry between 2003 and 2015, there was a 1.42-fold excess relative risk of malignancy compared to the age-adjusted population in the Surveillance, Epidemiology and End Results Program (SEER) database (1). The majority of malignancies were hematological, mainly lymphoid, and linked to the cell type affected by the PID. Significant increases in lymphoma in both males (10-fold) and females (8.34-fold) were observed. B-cell lymphomas are more common and there is an 8-fold increased risk for Non-Hodgkin’s Lymphoma (NHL) for all PIDs. Amongst DNA repair disorders, T- cell NHL is more common in ataxia telangiectasia (AT) whereas B-cell NHL is more common in Nijmegen Breakage Syndrome (NBS); malignancy is also the strongest negative factor affecting survival.

PIDs are categorized based on the segment of the immune system primarily involved, as shown in **Table 1**. Lymphoid malignancies are more common in all the categories except for congenital defects of stem cells or phagocytes, which are associated with increased risk of myeloid malignancies and myelodysplastic syndrome (5). We herein describe two cases of PIDs that manifested with a hematologic malignancy and discuss a diagnostic approach including when to suspect an underlying PID and diagnostic evaluation.

**Table 1:** Primary immune deficiencies and malignancy: pathophysiology, clinical presentation and diagnostic approach (2–4).

PID Category and Examples	Malignancy	Defective Mechanisms	Clinical Features	Investigations
**SCID/CID:** CARD11 deficiency DOCK8 deficiency ZAP70 deficiency CD40, CD40 ligand deficiencies	Lymphoma, B cell NHL	lymphocyte development co-signaling cytotoxicity tumor immunosurveillance	viral infections fungal infections bacterial infections opportunistic infections autoimmunity	PID diagnostic panel* T cell phenotyping Lymphocyte proliferation Rule out HIV Genetic testing
**CID with syndromic features:** cartilage hair hypoplasia Wiskott-Aldrich syndrome STAT3-HIES DiGeorge Syndrome	Lymphoma, *May be EBV+ with some*	lymphocyte development co-signaling cytotoxicity tumor immunosurveillance	viral, fungal, bacterial infections opportunistic infections autoimmunity	PID diagnostic panel* T cell phenotyping Lymphocyte proliferation Rule out HIV Genetic testing
		**Disease specific:** WAS: WASp-dependent rearrangements of the cytoskeleton and modulation of transcription and cell proliferation CHH: cell-mediated cytotoxicity and NK-like activity impaired	**Disease specific:** WAS: low level and small size of platelets CHH: short stature, short hair	**Disease specific:** WAS: flow cytometry for WASp Hyper IgE: Th17 flow cytometry DiGeorge: SNP microarray
**DNA repair defects/** **defects with radiation** **sensitivity:** ataxia telangiectasia Nijmegen breakage syndrome DNA Ligase IV deficiency Artemis	T-cell lymphoma, B-cell lymphoma, Leukemia	genetic instability	variable susceptibility to infections increased toxicity to chemotherapy Disease specific features: AT-ataxia and telangiectasias NBS- characteristic facial features	PID diagnostic panel* Chromosome breakage Radiosensitivity assays Genetic testing Disease specific: AT: alpha-fetoprotein NBS: chromosomal breakage
**Humoral defects:** Common variable immune deficiency (CVID) Activated p110δ syndrome Hyper IgM syndromes NFKB1 NFKB2	B cell lymphoma	co-signaling malignant transformation	recurrent sinopulmonary infections bronchiectasis lymphoproliferation autoimmunity	PID diagnostic panel* B cell phenotyping Pneumococcal vaccine challenge Genetic testing
**Primary immune regulatory disorders:** Autoimmune lymphoproliferative syndrome CTLA4 deficiency LRBA deficiency	Lymphoma	apoptosis tissue inflammation tumor surveillance	autoimmunity lymphoproliferation	PID diagnostic panel* Genetic testing Disease specific: ALPS: double negative T cells, ALPS panel CTLA4, LRBA: flow cytometry
**Defects with increased susceptibility to EBV induced lymphoproliferation:** Familial hemophagocytic lymphohistiocytosis X-linked lymphoproliferative disorder MAGT1 CD27, CD70, RASGRP1, CTPS1, CORO1A, CVID	Lymphoma, usually B cell and EBV positive	T and NK cell cytotoxicity tumor surveillance co-signalling	HLH lymphoproliferation chronic viral infections: EBV, HSV, HPV, warts autoimmunity	PID diagnostic panel* NK cell phenotyping CD107a/degranulation assays Invariant NKT cells Genetic testing Disease specific: Primary HLH: flow cytometry for perforin, SAP, XIAP expression
**Defects of Stem Cells and Phagocytes:** GATA2 Severe congenital neutropenia Shwachman Diamond Syndrome	myeloid malignancies, myelodysplastic syndrome	stem cell defects myeloid differentiation	neutropenia cytopenias bone marrow failure	Complete blood count with differential Bone marrow aspirate and biopsy PID diagnostic panel* Genetic testing
			**Disease specific** GATA2: monocytopenia, other organ features SDS: short stature, pancreatic insufficiency	**Disease specific:** SDS: fecal elastase

*PID diagnostic panel: lymphocyte subsets with T, B and NK cell enumeration, IgG, IgA, IgM, IgE levels, vaccine titers.

AFP, alpha fetoprotein; ALPS, autoimmune lymphoproliferative syndrome; AT, ataxia telangiectasia; CID, combine immune deficiency; CVID, common variable immune deficiency; DNT, double negative T cells, EBV, Epstein-Barr virus; FTT, failure to thrive; NGS, next generation sequencing; NHL, non Hodgkin’s lymphoma; PID, primary immunodeficiency; SCID, severe combined immune deficiency; SDS, Shwachman Diamond syndrome; WAS, Wiskott Aldrich syndrome; WES, whole exome sequencing; WGS, whole genome sequencing.

### Case #1

An 18-month-old boy presented with stridor and cyanosis with crying. Chest x-ray revealed an anterior mediastinal mass. Prior history was remarkable for rotavirus gastroenteritis requiring admission for 5 weeks following his second rotavirus vaccination, and two episodes of otitis media. Growth and development were normal. Parents were first cousins, and family history was notable for a cousin with leukemia at age 2.

Biopsy of the mass was consistent with stage III T-cell lymphoma. He was treated with standard protocol intermediate risk chemotherapy. His course was complicated by recurrent infections that included respiratory coronavirus HKU1 and rhinovirus infections, *Pseudomonas aeruginosa* and *candida* wound infections, neutropenic enterocolitis, presumptive lung fungal infection, and disseminated human simplex virus -1 infections. He also developed excess toxicity from chemotherapy: oral mucositis necessitating gastrostomy tube and prolonged neutropenia needing chemotherapy dose reduction. Immunological evaluation following therapy revealed normal T cell subsets, mitogen proliferation, B cell subsets, and immunoglobulins. T-cell repertoire was persistently abnormal and response to pneumococcal vaccination was poor. Alpha-fetoprotein was normal. Cancer predisposition next generation sequencing genetic panel revealed a homozygous c.7179T>G variant of unknown significance in the ATM gene. Chromosome breakage was highly suggestive of AT with 40% of cells with rearrangements involving chromosome 7 and 14. He developed ataxia at 30 months of age. The cousin with leukemia was subsequently found to have the same AT mutation.

### Case #2

A 4-year-old male with no past medical history presented with fever and lymphadenopathy. Imaging and biopsy showed stage III Epstein Barr virus (EBV)+ diffuse large B cell lymphoma. Family history was relevant for a maternal uncle that died of idiopathic liver failure in his 20’s. He was treated on a standard protocol and achieved full remission but presented again 6 years later with diffuse extranodal disease (stage IV), EBV+, including scalp and skin involvement. He underwent immune evaluation considering his family history, and extensive relapsed disease at a very young age. Evaluation revealed hypogammaglobulinemia with reduced memory B Cells and EBV PCR was 100,000 copies/mL in whole blood. SAP protein expression *via* flow cytometry was absent. Genetic testing through a PID panel revealed a mutation (c.23A>C,p.His8Pro) in SH2D1A confirming the diagnosis of type 1 X-linked lymphoproliferative syndrome (XLP). After achieving remission of his lymphoma, the patient underwent hematopoietic cell transplantation (HCT) as definitive cure.

## Diagnostic Approach

### When to Suspect PID

Clues in the clinical history and physical examination can aid in the diagnosis of an underlying PID. Early onset or recurrence of lymphoma should raise suspicion for an underlying PID, in particular DNA repair disorders such as AT and NBS. Presentation with a T-cell lymphoma or leukemia as an infant or toddler is a feature of AT (6). Patients with AT have telangiectasias that occur most frequently in the eyes (7). However, these features are usually not evident until 6 years of age (8). Often, ataxia is the first noticeable sign (9). Patients with NBS have distinctive facial features along with microcephaly and growth retardation (10). These features are usually apparent by 3 years of age. Additionally, increased toxicity from conventional chemotherapy as in Case #1 above should raise suspicion for an underlying DNA repair disorder (11).

A history of recurrent sinopulmonary infections or bronchiectasis is suggestive of an underlying humoral (B-cell) deficiency (12). A history of opportunistic infections such as *pneumocystis jiroveci* pneumonia or recurrent/chronic viral infections such as EBV or cytomegalovirus (CMV) infection favors a T-cell deficiency. Presence of severe warts or molluscum contagiosum are also concerning for an underlying T-cell defect such as DOCK8 deficiency. Eczema is also a common manifestation of T-cell disorders, including Wiskott Aldrich Syndrome (WAS), autosomal dominant STAT3 deficient Hyper IgE syndrome (STAT3-HIES) and DOCK8 deficiency (13). Autoimmunity is often a feature of PIDs, particularly primary immune regulatory disorders (PIRDs) (14). History of autoimmune cytopenia such as Evan’s syndrome or organ-specific autoimmunity such as type 1 diabetes or inflammatory bowel disease should raise concern for an underlying PID even without a history of recurrent infections (15).

EBV+ B-cell lymphoma can be a manifestation of PID, particularly those that have a high predisposition to EBV driven lymphoproliferative disorder (16). Examples include X-linked disorders such as X-linked lymphoproliferative disorder (XLP) and MAGT1 deficiency, and autosomal recessive disorders such as CD27 deficiency, CD70 deficiency, ITK deficiency, RASGRP1 deficiency, CTPS1 deficiency, CORO1A deficiency and DOCK8 deficiency. Proteins expressed by these genes are essential components of key pathways important for recognition of EBV-infected B cells by T cells and in the activation of the T- and NK-cell cytotoxicity responses toward EBV-infected B cells. Lymphoma is therefore more likely to be of B-cell origin. Common variable immune deficiency (CVID) can also be associated with EBV+ B-cell lymphoma. EBV+ B-cell lymphoma that is widespread at the time of diagnosis, has high histologic grades, and involves extra-nodal tissues, especially the gastrointestinal tract and central nervous system should prompt evaluation for an underlying PID. Other features that favor an underlying PID with a predisposition to EBV+ lymphoma include a history of pulmonary infections, hypogammaglobulinemia, and history of severe viral infections such as varicella, herpes simplex and CMV. Notably, most severe T-cell defects such as severe combined immune deficiency (SCID) tend not to present with EBV+ lymphoma since they develop very early-onset severe infections before they encounter EBV infection. Lastly, a family history of PID or onset of hematological malignancy in children or young adults is also an important clue for an underlying PID.

### How to Evaluate

**Figure 1:**
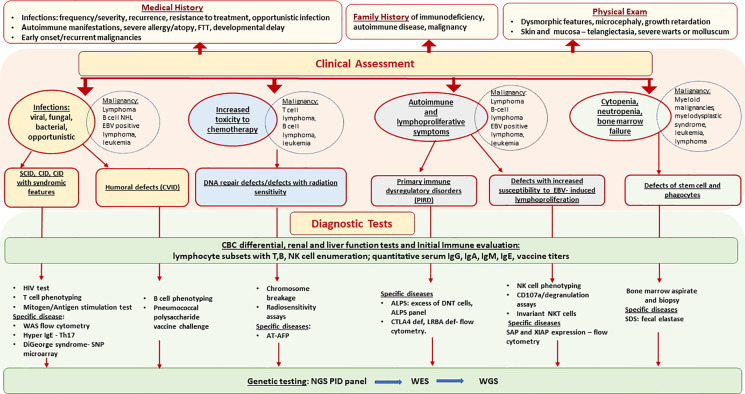
Primary Immunodeficiencies Associated with Hematologic Malignancy - Diagnostic Approach. AFP, alpha fetoprotein; ALPS, autoimmune lymphoproliferative syndrome; AT, ataxia telangiectasia; CID, combine immune deficiency; CVID, common variable immune deficiency; DNT, double negative cells, EBV, Epstein-Barr virus; FTT, failure to thrive; NGS, next generation sequencing; NHL, non Hodgkin’s lymphoma; SCID, severe combine immune deficiency; SDS, Schwachman Diamond syndrome; WAS, Wiskott Aldrich syndrome; WES, whole exome sequencing; WGS, whole genome sequencing.

Comprehensive evaluation that includes quantitative and qualitative assessment of both T-cell and B-cell immunity can aid in the diagnosis of an underlying PID as shown in **Figure 1** (17). The results must be compared with age-matched reference intervals as lymphocyte subsets and immunoglobulin levels vary with age. HIV infection must be ruled out in any patient being considered for a PID. Evaluation should include lymphocyte subsets to identify T-cell or B-cell lymphopenia and T-cell phenotyping based on the expression of cell-surface markers such as CD45RA and CD45RO to determine the proportion of naïve and memory CD4 and CD8 T-cells. Low proportion of naïve T-cells (CD45RA+) indicates a defect in T-cell differentiation and thymic output and favors a T-cell deficiency disorder such as AT, NBS, cartilage hair hypoplasia, SCID or combined immune deficiency. T-cell function can be evaluated by assessing proliferative responses to mitogens such as phytohemagglutinin, Concanavalin A or pokeweed and recall specific antigens such as Candida and tetanus toxoid. Impaired T-cell function favors a T-cell deficiency. Radiation sensitivity testing based on flow cytometric-based kinetic analysis of phosphorylated H2AX (γH2AX), ATM, and SMC1 in lymphocyte subsets, should be considered when a DNA repair disorder is suspected (18).

Evaluation of the B-cell arm of the immune system includes measuring the levels of the major immunoglobulin classes IgG, IgA, IgM and IgE. Measurement of specific antibody responses to prior vaccinations such as diphtheria, tetanus and pneumococcal vaccines is useful in identifying defective antibody production. B-cell phenotyping characterizing naïve, transitional, memory and class switched memory B-cells can help identify a defect in B-cell differentiation. Low immunoglobulin levels, non-protective vaccine antibody titers, decreased class switched memory B-cells are suggestive of a B-cell disorder such as CVID or CVID-like disorders such as LRBA deficiency and CTLA4 deficiency. Occasionally, patients with a B-cell disorder can present with a B-cell lymphoma prior to the development of hypogammaglobulinemia but may fail to recover B-cells or B-cell function following rituximab therapy. Hence, it is prudent to perform B-cell phenotyping in patients expected to receive rituximab therapy. In patients with severe allergic phenomena (eczema, eosinophilia, food allergies) measuring IgE levels can also be helpful in identifying certain disorders, i.e., Hyper IgE syndromes such as DOCK8 deficiency, STAT3-HIES and PGM3 deficiency.

Evaluation for autoimmune lymphoproliferative syndrome (ALPS) should be considered if there is a history of autoimmune cytopenia or chronic lymphadenopathy. An ALPS panel is a good initial screening test. Patients with ALPS usually have an increased proportion of T-cells expressing the alpha/beta T-cell receptor but lacking both CD4 and CD8 (α/β-double negative T- cells, aka DNTCs), increase in HLA-DR positive cells (on α/β DNTCs and CD8+ T-cells) with a concomitant loss/absence in CD25+ T-cells and reduced percentage of CD27+ B cells (2). Of note, elevation of DNTCs alone is not pathognomonic for ALPS and can be seen in other immune regulatory disorders.

In patients with EBV+ B-cell lymphoma with features discussed above that raise suspicion for underlying PID, evaluation should include quantification of immunoglobulin levels, B-cell phenotyping and analysis of invariant NK cells. Presence of hypogammaglobulinemia, reduced CD27+ memory B cells, and marked decrease of invariant NKT cells on flow cytometric analysis favors a PID with a unique predisposition to EBV associated lymphoma. Additionally, a history of HLH in boys should raise suspicion for either XLP and MAGT1 deficiency as inheritance is X-linked. SAP expression in T and NK cells should be evaluated by flow cytometric analysis in boys with EBV+ lymphoma or HLH. In patients with a history of HLH, NK-cell studies such as CD107a degranulation and NK-cell function can also aid in the diagnosis of an underlying PID.

Patients with a PID can manifest with a lymphoproliferative disorder (LPD), that can be difficult to distinguish from a lymphoma, either at initial presentation or recurrence. On histopathology, a polymorphic cell population favors a LPD. The presence of a monoclonal process on histology favors malignancy but clonal lymphocyte populations can also be seen in patients without lymphoma (19). Further evaluation with immunohistochemical and gene rearrangement studies are particularly helpful to determine cell lineage as well as to detect genetic/chromosomal aberrations (19).

If the history, physical examination, or immune evaluation are suggestive of an underlying PID, it can be helpful to categorize the type of PID (as shown in **Table 1**.). Genetic testing should be performed as the next step. Obtaining a genetic diagnosis in patients with PIDs is complex because more than 400 different PID-causing genes have been described. Additionally, many patients with similar genetic defects present with variable clinical and laboratory findings. Therefore, unless the evaluation provides an obvious clue to proceed with Sanger sequencing, for example, thrombocytopenia with small platelets and reduced T-cell WAS expression on flow cytometric analysis suggestive of WAS, next-generation sequencing (NGS) involving targeted PID panels is preferred (20). Many PID genes can be evaluated with a single test and current broad based NGS PID panels include >300 genes. Depth of coverage is often excellent and exonic deletions, which commonly occur in several PIDs, are successfully detected (21). If a genetic etiology is not identified despite targeted NGS testing, whole exome sequencing (WES) or whole genome sequencing (WGS) can be considered for second-line genetic testing. With increased accessibility and decreasing costs of testing, WES or WGS can also be considered as a first-line option. It is not uncommon for testing to identify novel variants of uncertain clinical significance (VUCS) in PID genes, making the diagnosis unclear. Further phenotypic and functional characterization can help determine whether the variant is pathogenic or benign.

A diagnosis of PID may guide future therapy and inform prognosis. Discovery of a genetic diagnosis can establish need for allogeneic HCT or identify a potential precision therapy such as leniolisib for activated phosphoinositide 3-kinase δ syndrome (3). In patients with a DNA repair disorder, malignancy is the strongest negative factor affecting survival. These patients would benefit from dose reduction in chemotherapy to limit toxicity. These patients are also at risk of developing future malignancies from imaging procedures that involve increased exposure to radiation such as CT scans and X-rays. Hence, these procedures need to be minimized and done only if absolutely necessary or opt for alternative imaging options such as MRI. Additionally, malignancy can recur in patients with an underlying PID and HCT is often indicated for definitive cure.

In conclusion, clinicians should be vigilant for an underlying PID in patients presenting with a hematologic malignancy. Onset of malignancy at an early age, especially of T-cell origin, a history of recurrent or opportunistic infections, autoimmunity or HLH, dysmorphic features on examination, growth retardation, increased toxicity during chemotherapy, or high grade EBV+ B-cell lymphoma with extranodal involvement should prompt immune evaluation for an underlying PID. NGS genetic testing should be considered early to facilitate the diagnosis of an underlying PID. Identification of an underlying monogenic PID provides important clinical benefits with the potential to alter therapy, impact prognosis and facilitate genetic counselling.

## Author Contributions

SC wrote the initial draft. TK created and edited the Table and Figure. NAMW and BJD each provided and wrote case summaries and contributed equally to expand the draft. All authors reviewed the complete paper and tables.

## Funding

NAMW and TK are supported by the Barb Ibbotson Chair in Pediatric Hematology, Alberta Children’s Hospital Foundation.

## Conflict of Interest

The authors declare that the research was conducted in the absence of any commercial or financial relationships that could be construed as a potential conflict of interest.

## Publisher’s Note

All claims expressed in this article are solely those of the authors and do not necessarily represent those of their affiliated organizations, or those of the publisher, the editors and the reviewers. Any product that may be evaluated in this article, or claim that may be made by its manufacturer, is not guaranteed or endorsed by the publisher.
